# Promotion of adipogenesis by neuropeptide Y during the later stages of chicken preadipocyte differentiation

**DOI:** 10.14814/phy2.13006

**Published:** 2016-11-02

**Authors:** Steven L. Shipp, Mark A. Cline, Elizabeth R. Gilbert

**Affiliations:** ^1^ Department of Animal and Poultry SciencesVirginia TechBlacksburgVA

**Keywords:** Adipogenesis, chick, differentiation, NPY, proliferation

## Abstract

Neuropeptide Y (NPY) promotes adipogenesis in both birds and mammals, although mechanisms in avians remain unclear. The objective of this study was thus to evaluate effects of NPY on chick preadipocyte proliferation and differentiation. Preadipocytes were treated with 0, 1, 10, or 100 nmol/L NPY and gene expression and cellular proliferation were evaluated at 12, 24, and 48 h. At 12 h posttreatment, mRNA abundance of topoisomerase II alpha (TOP2A), and thioredoxin‐dependent peroxidase 2 was upregulated and NPY was downregulated in response to NPY (0 vs. 100 nmol/L) in preadipocytes. Cells were also treated with NPY during differentiation and harvested at 8, 10, and 12 days postinduction of differentiation. At day 8 postinduction of differentiation, there was increased lipid accumulation (0 vs. 10 and 100 nmol/L), expression of CCAAT/enhancer binding protein *β* and fatty acid binding protein 4 (FABP4) (0 vs. 100 nmol/L), and sterol regulatory element‐binding protein (0 vs. 10 and 100 nmol/L) mRNA in NPY‐treated cells. The number of proliferating cells decreased on day 8 in response to NPY (0 vs. 10 nmol/L). At day 10, FABP4 and Kruppel‐like factor 7 mRNAs were downregulated (0 vs. 10 and 100 nmol/L, and 100 nmol/L, respectively), and at day 12, TOP2A mRNA was down‐regulated (0 vs. 100 nmol/L) in response to NPY treatment. Activity of glycerol‐3‐phosphate dehydrogenase (G3PDH) was increased on days 10 and 12 in NPY‐treated cells (0 vs. 100 nmol/L). Increased gene expression of proliferation markers in preadipocytes, and during differentiation increased expression of transcription factors and a fatty acid transporter, increased lipid accumulation, and increased activity of G3PDH suggest that NPY may enhance preadipocyte activity, adipogenesis, and promotes lipid accumulation throughout chicken adipocyte differentiation.

## Introduction

Neuropeptide Y (NPY) plays an important role in regulating whole‐body energy balance. Its orexigenic properties have been documented in numerous vertebrates, including mammalian (Clark et al. 1984; Pau et al. 1988), teleost (Matsuda et al. 2012), and avian species (Kuenzel et al. 1987; Richardson et al. 1995; Newmyer et al. 2013). NPY also promotes adipose tissue expansion in mice and humans (Kuo et al. 2007), with the adipogenic action of NPY most likely mediated via NPY receptor 2 (NPY2R) (Kuo et al. 2007). NPY also inhibits lipolysis in 3T3‐L1 cells, mediated by NPY1R (Park et al. 2014), and brown adipose tissue thermogenesis (Billington et al. 1991).

Our group recently showed that NPY influences proliferation and differentiation of adipose‐derived cells in chicks, the first report of the effects of an appetite‐regulatory peptide on adipose physiology in an avian species. We found that the stromal vascular fraction (SVF) of cells from the abdominal fat of young chicks respond to NPY with increased NPY and NPY2R mRNA expression at 4 h posttreatment (Zhang et al. 2015), suggesting that NPY promotes positive feedback of its own expression through NPY2R. There was also decreased expression (Zhang et al. 2015) of proliferation markers thioredoxin‐dependent peroxidase 2 (TPX2) (Brizova et al. 2010) and topoisomerase II alpha (TOP2A) (Milde‐Langosch et al. 2013). In that same study, we evaluated effects of daily NPY treatment on differentiation into adipocytes. After induction of differentiation, in response to NPY there was increased mRNA abundance of preadipocyte and proliferation markers until day 6 and decreased mRNA expression of transcription factors on days 4–6 postinduction of differentiation (Zhang et al. 2015). There was increased cellular proliferation on day 5 postinduction of differentiation in NPY‐treated cells, suggesting that NPY promotes mitotic expansion of chick preadipocytes during the initial stages of differentiation (Zhang et al. 2015). On day 6 postinduction, however, there was increased mRNA expression of lipoprotein lipase (LPL) and fatty acid binding protein 4 (FABP4) (Zhang et al. 2015), two factors important for cellular release and transport of fatty acids during adipogenesis (Cryer 1981; Amri et al. 1991; Cornelius et al. 1994), and increased activity of glycerol‐3‐phosphate dehydrogenase (G3PDH) (Zhang et al. 2015), an indirect marker of triacylglycerol synthesis (Swierczynski et al. 2003; Sledzinski et al. 2013). These data suggest that NPY enhances chick preadipocyte activity during the early stages of differentiation and promotes lipid deposition during the terminal stages of adipocyte maturation, although effects have not been reported after 12 h posttreatment in preadipocytes and after day 6 postinduction of differentiation.

Hence, to better understand NPY's effects on preadipocytes and during the later stages of adipocyte differentiation in chicken adipose cells, we evaluated gene expression and proliferation at 12, 24, and 48 h posttreatment with NPY, and cellular proliferation, G3PDH specific activity, neutral lipid accumulation, and gene expression on days 8, 10, and 12 postinduction of differentiation in cells treated with NPY during differentiation.

## Materials and Methods

### Animals

All animal protocols were approved by the Institutional Animal Care and Use Committee at Virginia Tech and animals were cared for in accordance with the Guide for the Care and Use of Laboratory Animals. Day of hatch Cobb‐500 broiler chicks were obtained from a local hatchery. Chicks were group caged at 30 ± 1°C and 50 ± 5% relative humidity with free access to water and a mash diet (22% crude protein and 3000 kcal metabolizable energy/kg), the composition of which has been reported elsewhere (Wang et al. 2015). The ambient temperature was gradually decreased from 30°C on day 1 to 25°C by 0.5°C per day, and then 25°C until 14 days posthatch.

### Primary adipose cell culture

Reagents were purchased from Sigma Aldrich (St. Louis, MO) unless otherwise stated. Approximately 2 g of abdominal fat was collected from 14‐day‐old male chicks by sterile dissection and submerged in DMEM/F12 Glutamax (Gibco, Grand Island, NY) media containing 1% penicillin/streptomycin (HyClone, Logan, UT) warmed to 37°C. Under the biological safety cabinet, adipose tissue was minced into fine sections with scalpel blades and incubated in 10 mL of 4‐(2‐hydroxyethyl) piperazine‐1‐ethanesulfonic acid (HEPES) solution (0.1 mol/L HEPES, 5 mmol/L d‐glucose, 1.5% bovine serum albumin; BSA) containing 500 units/mL Collagenase, Type I (Worthington Biochemical Corporation, Lakewood, NJ) for 1 h at 37°C in a shaking water bath. After the incubation, the contents were filtered through 250‐μm filters (Pierce, Rockford, IL). The filtrate was then centrifuged at 200 × *g* for 10 min to separate floating adipocytes from the other cell types. The supernatant was discarded, and cell pellets were resuspended in 10 mL of red blood cell lysis buffer (155 mmol/L NH_4_Cl, 10 mmol/L KHCO_3_, 0.1 mmol/L EDTA) to remove the red blood cells. The contents were then filtered through a 20‐μm mesh (Celltrics, Görlitz, Germany) to filter out the endothelial clumps. The filtrate was then centrifuged at 200 × *g* for 5 min to obtain the stromal vascular fraction (SVF) of cells. The SVF was resuspended in plating media (DMEM/F12 containing 10% defined fetal bovine serum (FBS); HyClone, and 1% penicillin/streptomycin) and seeded directly into a petri dish. After 72 h, cells were passaged one time, and then seeded 48 h later at a density of 3 × 10^4^ cells per mL into 12‐well plates (Falcon, Durham, NC) and incubated at 37°C in a 5% CO_2_ humidified atmosphere for at least 48 h (to minimize proinflammatory cytokine secretion) before beginning experiments. For all experiments, there were at least three biological replicates, where the experimental unit represented cells from an individual chick (*n* = 3 chicks), with triplicate wells of each treatment within an experiment for cells collected from a single chick. The triplicate values were averaged before statistical analysis.

### NPY treatment and cellular proliferation

Cells were seeded in 12‐well plates and cultured in plating media until 50% confluence. Cells were then cultured in serum‐free media for 24 h for cell cycle synchronization. In order to minimize the confounding effects of serum‐derived NPY, cells were cultured in basic media (DMEM/F12 with 1% penicillin/streptomycin) containing 1.5% FBS and 0, 1, 10, or 100 nmol/L chicken NPY (custom synthesized by AnaSpec; >98% purity).

The effect of NPY on cellular proliferation was evaluated using the Click‐iT^®^ EdU Alexa Fluor^®^ 488 Imaging Kit (Invitrogen, Carlsbad, CA). The EdU contains a nucleoside analog of thymidine and alkyne, which allows the thymidine analog to be incorporated into DNA during active DNA synthesis and be detected by the Alexa Fluor dye that contains the azide. Briefly, 1 μL of EdU was added into each well 2 h before the assay was conducted, a time recommended by the company to allow for adequate incorporation of EdU. Culture media were removed, and cells were fixed with 3.7% methanol‐free formaldehyde in phosphate‐buffered saline (PBS) for 15 min. Buffer containing 0.5% Triton X‐100 in PBS was then added to each well and cells were incubated for 20 min, followed by addition of 0.5 mL of Click‐iT reaction cocktail and incubation for 30 min. Cells were then stained with Hoechst 33342 solution for determining total cell number. Using a Nikon Eclipse Ti inverted microscope, representative images were captured and digitized with a charge‐coupled camera (SPOT, Diagnostic Imaging, Sterling Heights, MI), and analyzed using image overlay functions of NIS‐Elements Advanced Research Software (Nikon, Melville, NY). Briefly, five images were captured from different fields in each well for both of the fluorophores and image overlays performed. The proliferating cells and total cell number were counted for statistical analysis.

Neuropeptide Y was applied to cells at 8:00 am for the 24 and 48 h treatments and at 8:00 pm for the 12 h treatment in order to harvest cells at the same time of day. At 12, 24, and 48 h post‐NPY treatment, as well as days 8, 10, and 12 postinduction of differentiation, cells were fixed for the cellular proliferation assay and harvested for total RNA isolation and gene expression analysis of proliferation markers as described in the methods below.

### NPY treatment and cellular differentiation

The adipocyte differentiation protocol was similar to a method that has been described (Ramsay and Rosebrough 2003). Briefly, cells were cultured in plating media and allowed to reach complete confluence. Plating media (DMEM/F12 basic media with 10% FBS) was replaced with induction media (DMEM/F12 basic media containing 200 nmol/L insulin, 1 μmol/L dexamethasone, 10 U/mL heparin, and 2.5% chicken serum; CS) containing 0, 1, 10, or 100 nmol/L chicken NPY. At 48 h postinduction, media were replaced with insulin‐containing media (DMEM/F12 basic media containing 2.5% CS and 200 nmol/L insulin). After another 48 h, and for the remainder of the study, cells were cultured in maintenance media (DMEM/F12 basic media with 2.5% CS). Application of NPY treatment, the changing of media, and harvesting of cells occurred in the morning between the hours of 8:00 and 10:00 am.

### Oil Red O staining and lipid quantification

Cells were fixed with 10% neutral‐buffered formalin for 30 min at room temperature and Oil Red O staining was performed according to the manufacturer's instructions (Oil Red O Stain Kit; American Master Tech, Lodi, CA). Propylene glycol was added to each well and incubated for 5 min, replaced with preheated Oil Red O working solution and incubated for another 5 min at room temperature, and rinsed with distilled water. Cells were then counterstained with Modified Mayer's Hematoxylin (supplied in kit) for 1 min and rinsed with distilled water, then digital images captured to estimate the lipid content. To quantify neutral lipids, water was removed from each well, and 250 μL of 100% isopropanol was added to each well and incubated for 5 min to solubilize Oil Red O, and absorbance measured at 510 nm with a multimode plate reader (Tecan Infinite M200 Pro, Tecan, San Jose, CA) (Yang et al. 2013).

### Glycerol‐3‐phosphate dehydrogenase (G3PDH)‐specific activity

The method for assaying G3PDH‐specific activity was adapted from two other studies (Wise and Green 1979; Lengi and Corl 2010). Briefly, cells were cultured in 12‐well plates and treated as described above. On days 10 and 12 postinduction of differentiation, cells were washed with PBS, 200 μL of lysis buffer (50 mmol/L Tris–Cl, 1 mmol/L EDTA, and 1 mmol/L *β*‐mercaptoethanol, pH 7.5) was added to each well, and cells were detached from the plate using cell scrapers. Lysates were transferred to microcentrifuge tubes and further lysed with a 21‐gauge needle, then sonicated at 4°C using a Bioruptor 300 (Diagenode, Denville, NJ) with 4 cycles of 30 sec on and 30 sec off at high frequency. Lysates were then centrifuged at 12,000 × *g* at 4°C for 30 min, and the supernatant was used for measuring G3PDH activity and determining total protein concentration. The G3PDH activity was measured for each sample in duplicate in assay buffer (100 mmol/L triethanolamine–HCl, 2.5 mmol/L EDTA, 0.12 mmol/L NADH, 0.2 mmol/L dihydroxyacetone phosphate (DHAP), 0.1 mmol/L *β*‐mercaptoethanol, pH 7.5) in a total reaction volume of 200 μL in UV transparent plates (Corning, Durham, NC) using a *μ*Quant plate reader and KC Junior software (Bio‐Tek, Winooski, VT). Absorbance was measured at 340 nm for 20 cycles at 25°C and the maximum slope calculated from the absorbance data. Protein concentration was quantified with Bradford BCA reagent (Sigma‐Aldrich) using an Infinite M200 Pro multimode plate reader and Magellan software (Tecan). The maximum slope was normalized to the protein concentration to calculate specific activity, which is reported as μmol/min·mg.

### Total RNA isolation and real‐time PCR

Cells in 12‐well plates were washed with PBS and lysed with a 21‐gauge needle in 350 *μ*L RLT buffer (Qiagen, Valencia, CA). The total RNA was extracted with the RNeasy Mini kit (Qiagen) according to the manufacturer's instructions. The eluted total RNA samples were quantified and assessed for purity by spectrophotometry at 260/280/230 nm using a Nanophotometer^™^ Pearl (IMPLEN, Westlake Village, CA), and their integrity evaluated by agarose gel electrophoresis. The first‐strand cDNA was synthesized from 200 ng total RNA using the High Capacity cDNA Reverse Transcription kit (Applied Biosystems, Foster City, CA). Primers were designed in Primer Express 3.0 (Applied Biosystems; sequences reported in Zhang et al. (2015)). All primers were evaluated for amplification efficiency before use. Efficiency of target genes was within 5% of the reference gene (Actin). A total volume of 10 μL in each reaction contained 5 μL fast SYBR Green Master Mix (Applied Biosystems), 0.25 μL each of 5 μmol/L forward and reverse primers, and 3 μL of 10‐fold diluted cDNA. Real‐time PCR reactions were performed in duplicate for all samples on an Applied Biosystems 7500 FAST system, under the following conditions: enzyme activation for 20 sec at 95°C and 40 cycles of (1) melting step for 3 sec at 95°C and (2) annealing/extension step for 30 sec at 60°C. A melting curve analysis was performed after all reactions to ensure amplification specificity.

### Statistical analysis

The real‐time PCR data were analyzed using the ΔΔCT method, where ΔCT = CT target gene − CT Actin, and ΔΔCT = ΔCT target sample − ΔCT calibrator (Schmittgen and Livak 2008). The average of the control group within a time point was used as the calibrator sample. The relative quantity (2^−ΔΔCT^) values were subjected to ANOVA using the Fit Model Platform of JMP (SAS Inst., Cary, NC). The statistical model included the main effect of treatment. Tukey's test was used post hoc in all experiments, except for the proliferation study in which Dunnett's test was used to compare control and NPY‐treated groups for mRNA abundance of the preadipocyte and proliferation markers. A similar model was used for the cell count/percentage data, Oil Red O absorbance, and for G3PDH‐specific activity data. For the proliferation experiment, an arcsine transformation was applied to the percentage of proliferating cells before analysis. An additional analysis was performed on cell proliferation data for the proliferation and differentiation studies, where the statistical model included the main effect of time and Tukey's test was used post hoc to separate the means. Results were considered significant at *P *<* *0.05.

## Results

### Cellular proliferation

**Figure 1:**
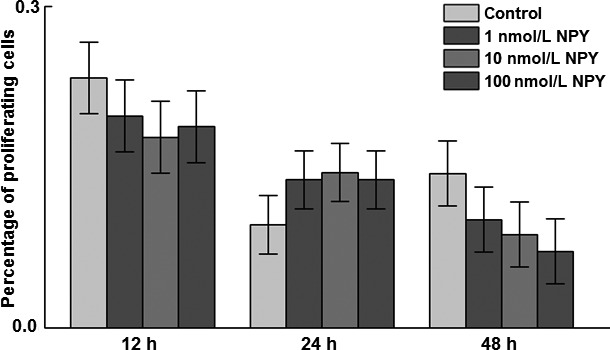
Effects of neuropeptide Y (NPY) treatment on proliferating cell number at 12, 24, and 48 h. The stromal–vascular fraction of cells isolated from 14‐day‐old chicken abdominal adipose tissue was treated with 0, 1, 10 or 100 nmol/L chicken NPY. Total cell number and number of proliferating cells were counted and the percentage of proliferating cells arcsine transformed before analysis. Values represent least squares means ± SEM of the percentage of proliferating cells (*n* = 3).

After reaching 50% confluence, cells were cultured in serum‐free media for 24 h for cell cycle synchronization and then in basic media containing 1.5% FBS and 0, 1, 10, or 100 nmol/L chicken NPY. There was no effect of NPY treatment on proliferating cell number or proliferating cells as a percentage of total cells at 12, 24, or 48 h posttreatment with NPY (Fig. 1). When the effect of time was included in the statistical model, the percentage of proliferating cells decreased from 12 to 24 and 48 h posttreatment, with no difference between 24 and 48 h posttreatment (*P *=* *0.0002; data not shown).

### Gene expression of preadipocyte and proliferation markers

**Figure 2:**
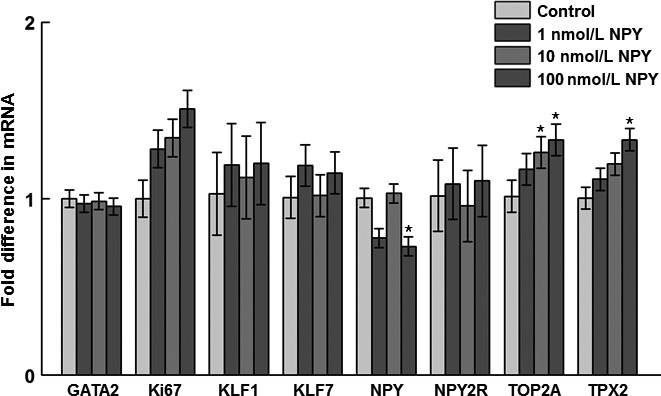
Effects of NPY treatment on gene expression of preadipocyte and proliferation markers at 12 h posttreatment in the stromal–vascular fraction of cells isolated from abdominal fat of 14‐day‐old broilers. GATA2: GATA‐binding protein 2 (*P *=* *0.98); Ki67: Ki67 (*P *=* *0.09); KLF1: Krüppel‐like factor 1 (*P *=* *0.79); KLF7: Krüppel‐like factor 7 (*P *=* *0.67); NPY: neuropeptide Y (*P *=* *0.02*); NPY2R: NPY receptor subtype 2 (*P *=* *0.88); TOP2A: topoisomerase II alpha (*P *=* *0.04*); TPX2: thioredoxin‐dependent peroxidase 2 (*P *=* *0.03*). Values represent least squares means ± pooled SEM (*n* = 3). Asterisk denotes a significant difference from the control (*P *<* *0.05; Dunnett's test).

At 12 h posttreatment with NPY, there was increased TPX2 mRNA in preadipocytes in response to 100 nmol/L NPY, increased TOP2A mRNA in response to both 10 and 100 nmol/L NPY, and decreased NPY mRNA in response to 100 nmol/L NPY (Fig. 2). There was no effect of NPY treatment on mRNA abundance of any of the genes measured at 24 or 48 h posttreatment with NPY (data not shown).

### Cellular proliferation during differentiation

**Figure 3:**
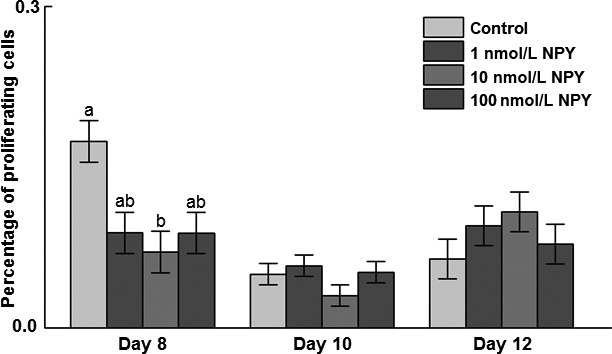
Effects of neuropeptide Y (NPY) treatment on cellular proliferation in chicken adipose cells at days 8, 10, and 12 postinduction of differentiation. The stromal–vascular fraction of cells was isolated from abdominal fat of 14‐day‐old broilers and upon confluence induced to differentiate in the presence of 0, 1, 10, or 100 nmol/L chicken NPY. At days 8, 10, and 12 postinduction of differentiation, cells were incubated in media containing 5‐ethynyl‐2 ´‐deoxyuridine (EdU) and stained at 2 h posttreatment with EdU. Cells were counted and the percentage of EdU‐positive cells was arcsine transformed and analyzed by ANOVA. Values represent least squares means ± SEM (*n* = 3). Different letters indicate a significant difference within day (*P *<* *0.05; Tukey's test).

On days 8, 10, and 12 postinduction of differentiation, cells were incubated with EdU 2 h before fixation and staining was performed to determine the number of proliferating cells. There was a decrease in the percentage of proliferating cells in response to 10 nmol/L NPY on day 8 (Fig. 3). There were no effects of NPY treatment on proliferation on day 10 or 12 postinduction of differentiation. When time was included in the statistical model, there was a decrease in the percentage of proliferating cells from days 8–10 and 12, but no difference between days 10 and 12 (*P *=* *0.006; data not shown).

### G3PDH‐specific activity during differentiation

**Figure 4:**
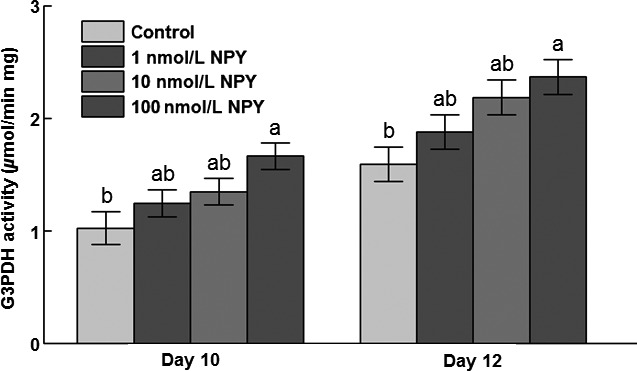
Specific activity of glycerol‐3‐phosphate dehydrogenase (G3PDH) at days 10 and 12 postinduction of differentiation in chicken abdominal adipose cells induced to differentiate in the presence of 0, 1, 10, or 100 nmol/L NPY. Values represent least squares means ± SEM (*n* = 3). Different letters indicate a significant difference within day (*P *<* *0.05; Tukey's test).

There was greater G3PDH activity in 100 nmol/L NPY‐ than control‐treated cells on both days 10 and 12 postinduction of differentiation (Fig. 4).

### Oil Red O staining and lipid accumulation during differentiation

**Figure 5:**
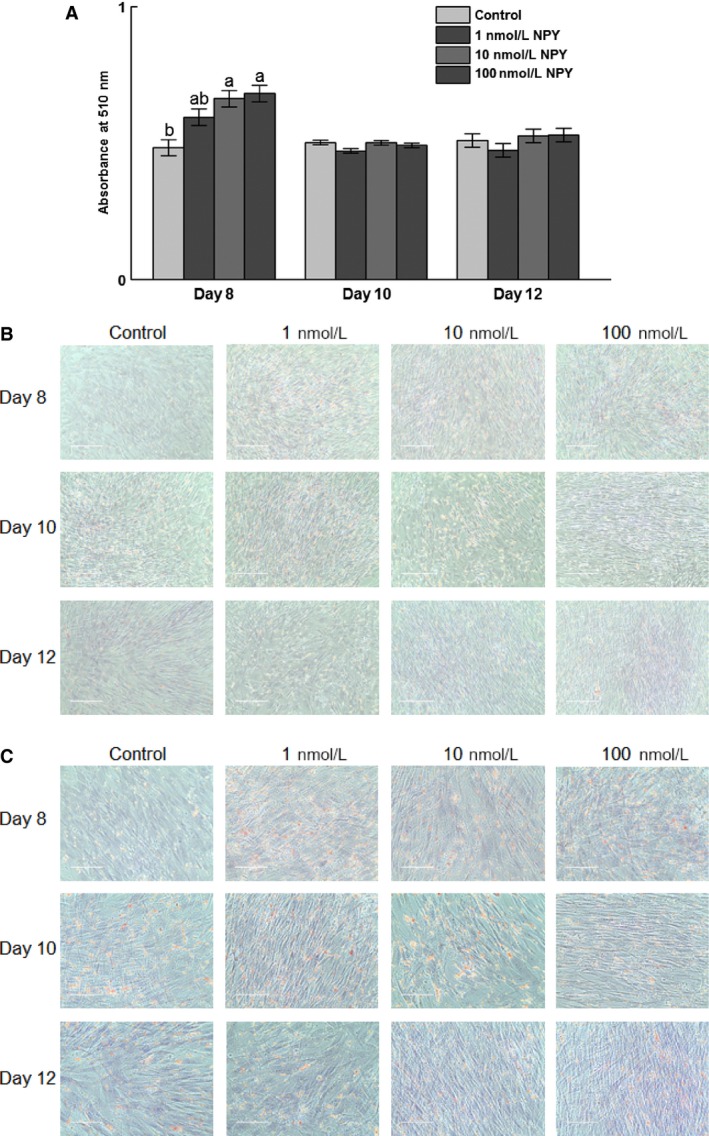
Oil Red O staining of chicken adipose cells treated with neuropeptide Y (NPY). The stromal–vascular fraction of cells was isolated from abdominal fat of 14‐day‐old broilers and upon confluence induced to differentiate in the presence of 0, 1, 10, or 100 nmol/L chicken NPY. At 8, 10, and 12 days postinduction of differentiation cells were stained with Oil Red O and counterstained with Modified Mayer's Hematoxylin and images captured. (A) Absorbance at 510 nm on days 8, 10, and 12 postinduction of differentiation. (B) Representative micrographs of 0, 1, 10, and 100 nmol/L NPY‐treated cells on days 8, 10, and 12 postinduction of differentiation under 20× magnification. (C) Representative micrographs of 0, 1, 10, and 100 nmol/L NPY‐treated cells on days 8, 10, and 12 postinduction of differentiation under 40× magnification. The scale bar indicates 200 and 100 μm for (B) and (C), respectively. The red color indicates the staining of neutral lipids. Values represent absorbance least squares means ± SEM (*n* = 3). Different letters indicate a significant difference within day (*P *<* *0.05; Tukey's test).

There was increased lipid accumulation (absorbance of Oil Red O at 510 nm) at day 8 postinduction of differentiation (*P *=* *0.006) in response to 10 and 100 nmol/L NPY (Fig. 5A). There was no effect of treatment on absorbance at day 10 (*P *=* *0.09) or 12 (*P *=* *0.43) postinduction of differentiation (Fig. 5A). Representative micrographs are shown in Figure 5B and C.

### Gene expression during differentiation

**Figure 6:**
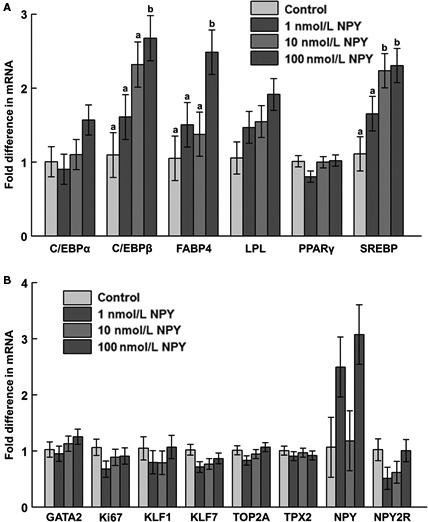
The mRNA abundance of (A) adipogenesis‐associated factors, (B) preadipocyte and proliferation markers, and neuropeptide Y (NPY) and NPY receptor 2 (NPY2R) at day 8 postinduction of differentiation in chicken adipose cells treated with 0, 1, 10, or 100 nM chicken NPY. (A) C/EBP *α*: CCAAT/enhancer binding protein *α* (*P *=* *0.18); C/EBP *β*: CCAAT/enhancer binding protein *β* (*P *=* *0.03*); FABP4: fatty acid binding protein 4 (*P *=* *0.04*); LPL: lipoprotein lipase (*P *=* *0.12); PPAR *γ*: peroxisome proliferator‐activated receptor *γ* (*P *=* *0.21); SREBP: sterol regulatory element‐binding protein (*P *=* *0.02*); (B) GATA2: GATA‐binding protein 2 (*P *=* *0.48); Ki67: Ki67 (*P *=* *0.37); KLF1: Krüppel‐like factor 1 (*P *=* *0.67); KLF7: Krüppel‐like factor 7 (*P *=* *0.19); TOP2A: topoisomerase II alpha (*P *=* *0.26); TPX2: thioredoxin‐dependent peroxidase 2 (*P *=* *0.81); NPY (*P *=* *0.07); NPY2R (*P *=* *0.22). Values represent least squares means ± pooled SEM (*n* = 3). Bars with different letters within a gene represent a significant difference (*P *<* *0.05; Tukey's test).

**Figure 7:**
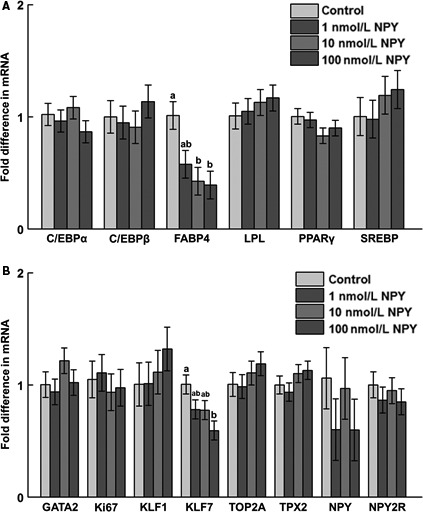
The mRNA abundance of (A) adipogenesis‐associated factors, (B) preadipocyte and proliferation markers, and neuropeptide Y (NPY) and NPY receptor 2 (NPY2R) at day 10 postinduction of differentiation in chicken adipose cells treated with 0, 1, 10, or 100 nmol/L chicken NPY. (A) C/EBP *α*: CCAAT/enhancer binding protein *α* (*P *=* *0.51); C/EBP *β*: CCAAT/enhancer binding protein *β* (*P *=* *0.71); FABP4: fatty acid binding protein 4 (*P *=* *0.03*); LPL: lipoprotein lipase (*P *=* *0.76); PPAR *γ*: peroxisome proliferator‐activated receptor *γ* (*P *=* *0.35); SREBP: sterol regulatory element‐binding protein (*P *=* *0.62); (B) GATA2: GATA‐binding protein 2 (*P *=* *0.40); Ki67: Ki67 (*P *=* *0.88); KLF1: Krüppel‐like factor 1 (*P *=* *0.64); KLF7: Krüppel‐like factor 7 (*P *=* *0.05); TOP2A: topoisomerase II alpha (*P *=* *0.53); TPX2: thioredoxin‐dependent peroxidase 2 (*P *=* *0.36); NPY (*P *=* *0.54); NPY2R (*P *=* *0.77). Values represent least squares means ± pooled SEM (*n* = 3). Bars with different letters within a gene represent a significant difference (*P *<* *0.05; Tukey's test).

**Figure 8:**
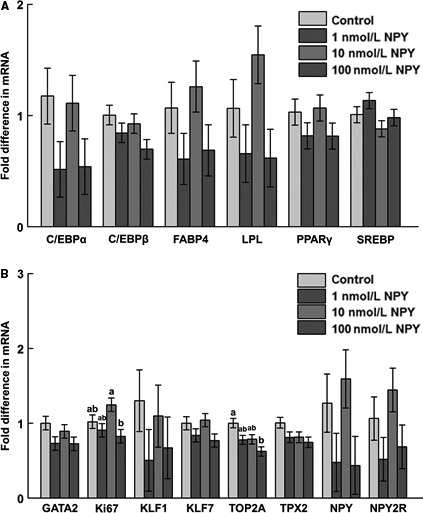
The mRNA abundance of (A) adipogenesis‐associated factors, (B) preadipocyte and proliferation markers, and neuropeptide Y (NPY) and NPY receptor 2 (NPY2R) at day 12 postinduction of differentiation in chicken adipose cells treated with 0, 1, 10, or 100 nmol/L chicken NPY. (A) C/EBP *α*: CCAAT/enhancer binding protein *α* (*P *=* *0.19); C/EBP *β*: CCAAT/enhancer binding protein *β* (*P *=* *0.16); FABP4: fatty acid binding protein 4 (*P *=* *0.22); LPL: lipoprotein lipase (*P *=* *0.11); PPAR *γ*: peroxisome proliferator‐activated receptor *γ* (*P *=* *0.33); SREBP: sterol regulatory element‐binding protein (*P *=* *0.19); (B) GATA2: GATA‐binding protein 2 (*P *=* *0.17); Ki67: Ki67 (*P *=* *0.04*); KLF1: Krüppel‐like factor 1 (*P *=* *0.53); KLF7: Krüppel‐like factor 7 (*P *=* *0.16); TOP2A: topoisomerase II alpha (*P *=* *0.02*); TPX2: thioredoxin‐dependent peroxidase 2 (*P *=* *0.13); NPY (*P *=* *0.16); NPY2R (*P *=* *0.19). Values represent least squares means ± pooled SEM (*n* = 3). Bars with different letters within a gene represent a significant difference (*P *<* *0.05; Tukey's test).

On day 8 postinduction of differentiation, there was increased mRNA abundance of C/EBP*β* and FABP4 in response to 100 nmol/L NPY, and upregulation of SREBP mRNA in response to 10 and 100 nmol/L NPY (Fig. 6A). On day 10, there was decreased mRNA abundance of FABP4 mRNA in response to 10 and 100 nmol/L NPY (Fig. 7A), and decreased mRNA abundance of KLF7 in response to 100 nmol/L NPY (Fig. 7B). On day 12 postinduction of differentiation there was decreased TOP2A mRNA in response to 100 nmol/L NPY, and decreased expression of Ki67 in response to 100 as compared to 10 nmol/L NPY (Fig. 8B).

## Discussion

### Effects of NPY on adipose cell proliferation

To understand NPY's effects on growth of the precursor cells isolated from the SVF of chick adipose, we evaluated cellular proliferation and gene expression at several time points posttreatment in precursor cells grown under conditions to promote proliferation. In our earlier study, effects on gene expression were measured at 4 h and cellular replication at 12 h posttreatment with NPY (Zhang et al. 2015). The number of proliferating cells was not affected by NPY at 12 h posttreatment in the previous study (Zhang et al. 2015). To evaluate whether this was sufficient time for effects of NPY to be observed as transcriptional or cell number changes, this study was extended to include 12, 24, and 48 h as the end points. In our previous study, at 4 h posttreatment with NPY, there was decreased TPX2 and TOP2A mRNA (Zhang et al. 2015), the opposite of what we observed in this study at 12 h posttreatment. Because expression of these genes decreased at 4 h, but increased at 12 h, the increase at 12 h may not be a direct response to NPY, but rather recovering from the decrease in expression observed at 4 h to reestablish normal growth. Also, at 4 h, expression of NPY and NPY2R increased (Zhang et al. 2015), whereas at 12 h NPY mRNA decreased in response to the same dose. This could be due to a similar mechanism as for TPX2 and TOP2A; that the decrease in NPY at 12 h may not be a direct response to NPY, but rather a recovery to normal homeostatic levels of expression. This is further evidenced by the absence of changes in expression at 24 and 48 h. The direct effects of NPY on the SVF of cells may occur earlier than 12 h. Because of the lack of an effect on cell proliferation at any of the time points measured in either study (12, 24, or 48) it is likely that NPY does not act to enhance cell proliferation in adipose precursor cells in chickens. However, that some transcriptional effects were observed early on during treatment suggests that NPY is affecting metabolic activity of the cells.

The percentage of proliferating cells decreased from 12 to 24 h postexposure to growth media after serum deprivation, likely because the total number of cells amplified with time. That percentages did not differ between 24 and 48 h suggests that a maximal level of proliferation under the culture conditions had been reached by 24 h. The percentage of proliferating cells also differed among days 8, 10, and 12 postinduction of differentiation. This is likely because with time a greater number of cells became terminally differentiated and incapable of proliferating.

In 3T3‐L1 preadipocytes, low doses of NPY (10^−15^ and 10^−13^ mol/L) enhance proliferation after 24 h, but higher doses had no effect (Tang et al. 2015). This study did not evaluate an NPY dose lower than 1 nmol/L (10^−9^), thus it is possible that a lower dose would produce an effect on proliferation. An effect with a dose that low would be unexpected though, as the lowest dose used in our studies had no effect on mRNA abundance of any of the genes measured at any time point (4, 12, 24, and 48 h) in our previous (Zhang et al. 2015) and present study.

### Effects of NPY on adipose cell differentiation

During differentiation, PPAR*γ*, C/EBP *α* and *β*, and SREBP are important transcription factors that are sequentially induced and function to regulate expression of factors that are involved in lipid accumulation and expansion of the adipocyte, as reviewed (Rosen et al. 2000; Rosen and MacDougald 2006). In our previous study, PPAR*γ*, C/EBP*α*, and SREBP mRNAs decreased in response to NPY on day 4 postinduction of differentiation (Zhang et al. 2015). Expression of preadipocyte and proliferation markers TOP2A, TPX2, GATA2, and Ki67 increased in response to NPY throughout the first 6 days postdifferentiation (Zhang et al. 2015). Taken together, this may suggest that NPY is delaying differentiation by inhibiting expression of these transcription factors important for development of the adipocyte in order to increase preadipocyte proliferation and activity during the early stages of adipogenesis (Zhang et al. 2015). That there was increased expression of two transcription factors on day 8, C/EBP*β* and SREBP, in this study, further supports that the effect of NPY during differentiation may be to increase preadipocyte activity early on and delay differentiation until the later stages, where transcription factors were observed to increase in this study. SREBP has also been reported to cause chicken embryo fibroblasts to transdifferentiate into adipocyte‐like cells when expressed by retrovirus‐mediated gene transfer (Liu et al. 2010), showing its importance during adipocyte differentiation and suggesting that in this study increased expression on day 8 contributes to adipocyte differentiation. There was also decreased expression of KLF7 on day 10 and TOP2A on day 12 in response to NPY, possibly due to the increased adipogenic activity and fewer preadipocyte cells at this stage of differentiation. Another explanation for the decreased expression of TOP2A on day 12 could be that there was apoptosis of cells that were unable to fully differentiate.

On day 5 postinduction of differentiation there was increased proliferation in NPY‐treated cells (Zhang et al. 2015), an effect that may have been due to the presence of preadipocytes still undergoing differentiation that were either induced to proliferate or to undergo the mitotic expansion that is characteristic of mammalian 3T3L‐1 preadipocytes (Patel and Lane 2000). To better understand this unexpected effect, we measured cellular proliferation on days 8, 10, and 12 postinduction, times at which a greater proportion of the cells should be terminally differentiated. That there was no increase in proliferation during the later time points suggests that the effect on proliferation at day 5 is from cells that have not yet terminally differentiated into adipocytes. This is further evidenced by the decrease in proliferation on day 8, and combined with gene expression data suggests that transcriptional regulation has switched from increasing preadipocyte activity to increasing adipogenic activity. The cell proliferation results support that more cells were terminally differentiated at days 10 and 12 postinduction, as there were fewer proportions of proliferating cells and mature adipocytes are thought to be incapable of division (Hausman et al. 2001). These data support that our experimental model is representative of preadipocyte differentiation, and that by day 10 most cells were terminally differentiated.

At day 6 postinduction of differentiation, G3PDH activity was greater in NPY‐treated cells (Zhang et al. 2015), suggesting that NPY affects triacylglycerol synthesis either directly or indirectly as an important component of terminal differentiation (Swierczynski et al. 2003; Sledzinski et al. 2013). In this study this effect persisted through days 10 and 12, suggesting that NPY continued to have an effect on terminal differentiation through the later stages of adipocyte maturation. That there was no change in G3PDH activity during the first 4 days postinduction of differentiation in our earlier study (Zhang et al. 2015), but a clear effect on days 6–12, suggests that NPY treatment is promoting fat synthesis during the later stages of differentiation. This is further supported by the increase in neutral lipid accumulation at day 8, an effect also observed in 3T3‐L1 cells at day 8 of differentiation (Tang et al. 2015). Decreased expression of FABP4 on day 10 may suggest that elevated levels of this specific binding protein are no longer needed past day 10 in the lipid droplet‐containing adipocyte, but that expression of FABP4 increased on days 2, 6, and 8 suggests that NPY signaling is associated with increasing the amount of fatty acids available in the cell to be transported to assemble the triacylglycerols in adipocytes.

In conclusion, results suggest that NPY increases chick preadipocyte activity within the first 12 h posttreatment, but has no effect on increasing proliferation under the conditions used in the present study. During chick preadipocyte differentiation, NPY treatment may be associated with enhanced preadipocyte activity during the early phase, thereby delaying differentiation until the terminal stages, where increased expression of SREBP, C/EBP*β*, and FABP4, increased lipid accumulation, increased G3PDH activity, and decreased expression of KLF7 and TOP2A all support that there was greater adipogenic activity. Understanding the mechanisms underlying effects of appetite‐regulating peptides on physiological pathways associated with energy metabolism in the periphery in an avian model may have implications for understanding metabolic disorders in humans.

## Conflict of Interest

None declared.
